# Network targets for therapeutic brain stimulation: towards personalized therapy for pain

**DOI:** 10.3389/fpain.2023.1156108

**Published:** 2023-06-08

**Authors:** Julian C. Motzkin, Ishan Kanungo, Mark D’Esposito, Prasad Shirvalkar

**Affiliations:** ^1^ Departments of Neurology and Anesthesia and Perioperative Care (Pain Management), University of California, San Francisco, San Francisco, CA, United States; ^2^ Department of Neurological Surgery, University of California, San Francisco, San Francisco, CA, United States; ^3^ Department of Psychology, University of California, Berkeley, Berkeley, CA, United States

**Keywords:** neuromodulation, chronic pain, network neuroscience, graph theory, precision medicine, pain, deep brain stimulation (DBS), transcranial magnetic stimulation (TMS)

## Abstract

Precision neuromodulation of central brain circuits is a promising emerging therapeutic modality for a variety of neuropsychiatric disorders. Reliably identifying in whom, where, and in what context to provide brain stimulation for optimal pain relief are fundamental challenges limiting the widespread implementation of central neuromodulation treatments for chronic pain. Current approaches to brain stimulation target empirically derived regions of interest to the disorder or targets with strong connections to these regions. However, complex, multidimensional experiences like chronic pain are more closely linked to patterns of coordinated activity across distributed large-scale functional networks. Recent advances in precision network neuroscience indicate that these networks are highly variable in their neuroanatomical organization across individuals. Here we review accumulating evidence that variable central representations of pain will likely pose a major barrier to implementation of population-derived analgesic brain stimulation targets. We propose network-level estimates as a more valid, robust, and reliable way to stratify personalized candidate regions. Finally, we review key background, methods, and implications for developing network topology-informed brain stimulation targets for chronic pain.

## 1. Introduction

The Centers for Disease Control and Prevention (CDC) estimates that chronic pain affects more people in the United States than heart disease, diabetes and cancer combined. Approximately 20% of the United States adult population suffers with chronic pain, with more than 19 million experiencing functionally disabling “high impact” chronic pain that limits life, work and social activity (1). Patients with chronic pain have highly variable and often inadequate responses to treatment, leading to trial-and-error based interventions that delay relief and increase reliance on potentially addictive opioid analgesics. Beginning in the 1950s, an emerging understanding of the role of specific brain regions in pain transmission and modulation motivated trials of deep brain stimulation (DBS) for severe and otherwise refractory pain conditions of various etiologies. More recently, preclinical pain research and neural circuitry models of pain processing in humans have substantially broadened the range of viable empirically-defined targets that may be used to treat chronic pain (2, 3). Although DBS of a variety of specific brain regions can dramatically reduce pain and improve quality of life for a subset of patients, therapeutic responses to stimulation are highly variable and there are currently no reliable methods for determining optimal stimulation parameters and locations for individual patients. The emergence of clinical transcranial magnetic stimulation (TMS) in the 1990s and more recent development of MRI-guided low-intensity focused ultrasound (LI-FUS) offer promising non-invasive alternatives to DBS (4–7). These new approaches promise to improve the safety and accessibility of therapeutic brain stimulation for pain, but face similar challenges in identifying in whom, where, and how to stimulate to achieve optimal pain relief.

Dysfunction in distributed brain networks is increasingly recognized as central to the pathophysiology of a variety of neuropsychiatric disorders and approaches to modulate these networks are now actively investigated as novel therapies (8–12). Across disorders, therapeutic targets are generally established anatomical regions informed by prior DBS and/or preclinical models, or regions identified through population-level comparisons between patients with the disorder and healthy adult “control” cohorts (8, 11–14). Although improving the precision of stimulation by targeting specific neuroanatomical regions that are abnormally activated or connected at the population level has been linked to improved treatment outcomes for a variety of disorders, there is more limited evidence that such an approach can be prospectively applied to individual patients (9, 15–17). In fact, there is accumulating evidence across neuropsychiatric disorders that variability in clinical response to neuromodulation is directly related to variability in the *topology* (i.e., the spatial organization and arrangement of network connections) of underlying brain networks (18–21).

Advances in precision neuroscience have recently enabled the estimation of reliable individual brain networks by repeatedly scanning research participants (22–25). Studies of these highly sampled individuals show that networks exhibit substantial interindividual variability and that individual networks meaningfully diverge from population average estimates (26–31). Moreover, neural responses to both acute experimental pain and spontaneous fluctuations in chronic pain are highly variable across individuals (32–34). Indeed, population-level brain imaging studies have largely failed to capture the complex and uniquely individual aspects of pain. This failure poses a considerable translational barrier to the development of network-informed biomarkers that can be used guide personalized neuromodulation for chronic pain (35, 36).

Effective pain management demands a personalized approach. We propose that a precision neuromodulation approach to network-informed target selection will be more robust to individual differences in brain network topologies than current one-target-fits-all approaches. Here, we review the neuroanatomical basis of pain in the context of stimulation for clinical pain relief and critique existing methods used to identify optimal targets for therapeutic neuromodulation. We conclude with a proposed biomarker development strategy motivated by recent advances in network neuroscience and graph theory that may improve personalized stimulation for pain and other neuropsychiatric disorders.

## 2. Review of pain circuits

Research across species indicates that pain is represented by a distributed network of brain regions involved in different aspects of pain processing, including ascending pain transmission circuits and descending opioidergic pain modulation circuits (32, 37–43). Early attempts to identify a single locus of pain perception in humans converged on the thalamus, as neither focal lesions nor stimulation of cortex could reliably modify painful sensations (44, 45) [but see (46, 47)]. Gate Control Theory (48) offered a mechanistic explanation for variability in pain perception as a dynamic central processes that exerts top-down influence over the patterned input of spinal pain transmission pathways. Gate control theory also began to highlight the paramount importance of motivational and affective features of the pain experience (49). This emerging understanding of pain as a multidimensional psychological construct was later formalized by Melzack as the Neuromatrix, subsequently “Pain Matrix”, a widely distributed neural network of interconnected somatosensory, limbic, and thalamocortical structures subserving parallel processing of sensory-discriminative, affective-motivational, and cognitive-evaluative domains of the pain experience (50, 51). In contrast to the largely modulatory role ascribed to the brain in Gate Control Theory, the Neuromatrix is foundational to our current understanding of pain as a complex experience emergent from patterned activity across a distributed network of brain regions (52).

**Figure 1:**
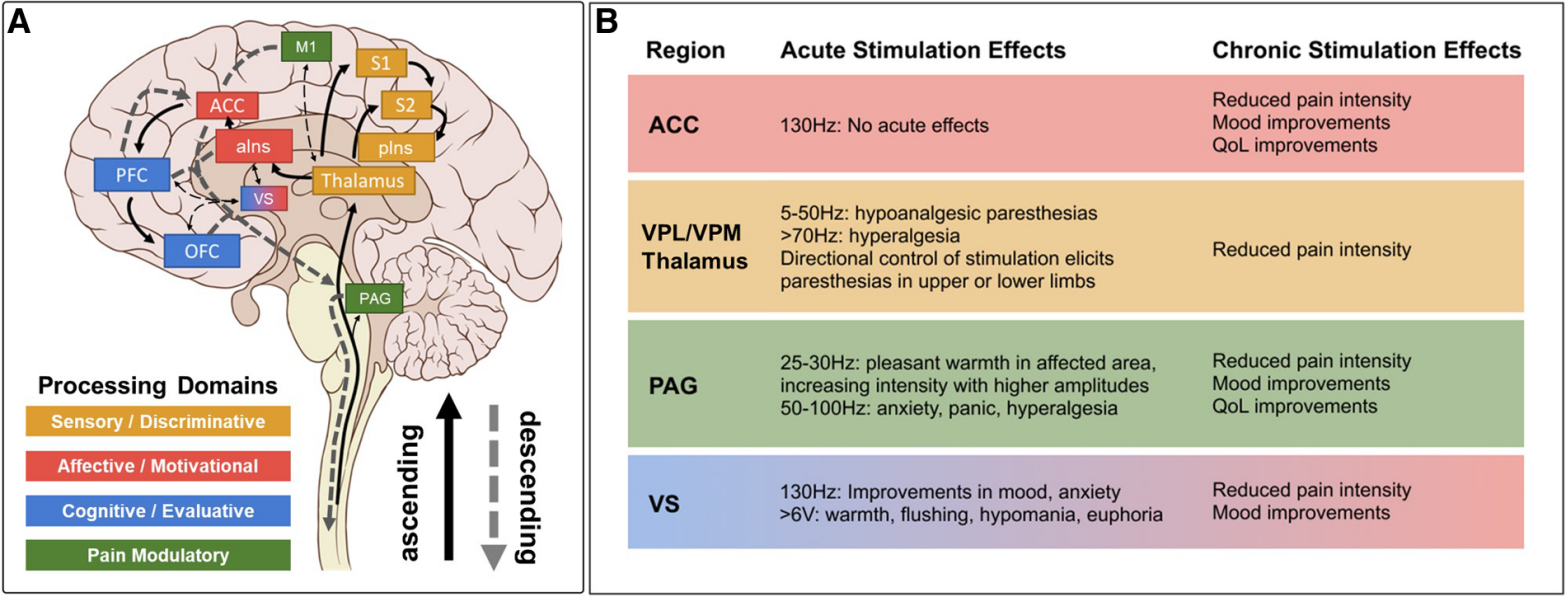
Pain matrix regions grouped by processing domain* with summarized DBS effects. (**A**) Brain regions implicated in different domains of pain perception superimposed on ascending pain transmission and descending pain modulation pathways. Several implicated brain regions without stimulation data in chronic pain (e.g., amygdala, hippocampus) are excluded for clarity, as are many connections between regions. Solid arrows indicate ascending projections. Dashed arrows indicate descending projections. (**B**) Summary of acute and chronic effects of stimulation at established DBS targets, color coded by processing domain as in **A**, with notes regarding specific parameter effects. Stimulation effects on relevant domains are summarized from references cited in Tables 1, 2. Clinical efficacy summaries can be found in Table 3. PAG, periaqueductal gray; S1, primary somatosensory cortex; S2, secondary somatosensory cortex; pIns, posterior insula; M1, primary motor cortex; aIns, anterior insula; ACC, anterior cingulate cortex; VS, ventral striatum; PFC, prefrontal cortex; OFC, orbitofrontal cortex; VPL, ventral posterolateral; VPM, ventral posteromedial; QoL, quality of life. *Note that we operationally define the term “domain” as a collection of related psychological processes or functions (e.g., somatosensation, emotion, cognition, memory, etc.) for consistency with the psychology and cognitive neuroscience literature (cited from Shirvalkar et al., (3)).

Modern accounts of the Pain Matrix predict that sensory, affective, and cognitive domains of pain processing are represented by a specific subset of established “pain relevant” brain regions (51, 53, 54) (Figure 1A). In this reductionist view, sensory-discriminative regions include the sensory (i.e., ventral posterolateral and ventral posteromedial) thalamus, somatosensory cortex (S1/S2), and posterior insula (pIns). Affective-motivational regions include the amygdala, ventral striatum (VS), anterior insula (aIns), and anterior cingulate cortex (ACC). Cognitive-evaluative regions include the hippocampus, orbitofrontal cortex (OFC), and dorsolateral prefrontal cortex (dlPFC). Subcortical structures like the sensory thalamus and periaqueductal gray (PAG) with extensive reciprocal connections to distributed cortical regions and downstream outputs that are known to modulate spinal nociceptive transmission are included in most models as key ascending transmission and descending modulation hubs, respectively. Below, we review neuroimaging evidence for and against the involvement of specific brain regions and networks in pain before considering implications for clinical neuromodulation.

**Table 1:** Stimulation parameters and response rates for prior DBS studies targeting chronic pain.

	Pain subtype (# patients)	Stimulation parameters	Response rate (pain reduction in cohort; pain survey administered)	Follow up time	Type of study	References
Single target						
VPL/VPM	Neuropathic pain (5)	100–120 Hz, 1–1.5 mA	80% (NR)	Range 3–24 months	Case series	(153)
	Neuropathic pain (16)	200–450 µs pulse width, 5–50 Hz, 0.5–5 V	NR (52.8% median; VAS)	36 months	Case series	(154)
	Neuropathic pain (12)	200–450 µs, 5–50 Hz, 0.5–5 V	NR (69.6% average; VAS)	12 months	Case series	(155)
	Neuropathic pain (10)	NR	60% (>80%; VAS)	Range 2–18 years	Case series	(156)
	Neuropathic pain (1)	100 µs pulse width, 99 Hz, 0.1 mA, cycling: 30 s ON, 10 s OFF	100% (50%; VAS)	16 months	Case report	(311)
	Neuropathic pain (18)	150–210 µs pulse width, 20–135 Hz	73% (NR)	NR	Case series	(157)
PAG/PVG	Neuropathic pain (2)	NR	100% (62.5% average; VAS)	Average 16 months	Case series	(158)
	Neuropathic pain (16)	150–450 µs pulse width, 5–80 Hz, 2.5–5 V	NR	Average 20.6 months	Case series	(159, 160)
	Neuropathic pain (4)	25–75 Hz, 2–5 V	NR	Right after stimulation	Case series	(161)
	Neuropathic pain (4)	120–210 µs pulse width, 25–30 Hz, 2.8–3.5 V	75% (NR)	up to 31 months	Case series	(162)
VS/ALIC	Neuropathic pain (10)	60–210 µs pulse width, 130 Hz, 1–6 V	11% (>50%; VAS)	24 months	Double-blind, randomized, placebo controlled, crossover	(163)
PLIC	Neuropathic pain (4)	60–150 µs pulse width, 20–60 Hz, 1–4.5 V	75% (>40%; VAS)	12 months	Case series	(164)
ACC	Neuropathic pain (2)	130 Hz	100% (NR)	4 months	Case series	(165)
	Neuropathic pain (16)	450 µs pulse width, 130 Hz, 4–6.5 V	33% (24.5% average; VAS)	Average 13.2 months	Case series	(166)
	Neuropathic pain (24)	450 µs pulse width, 130 Hz, 4–6.5 V	45.5% (60.3% average; NRS)	6 months	Case series	(167)
	Neuropathic pain (9)	450 µs pulse width, 130 Hz, 4–5.5 V	NR (37.9% average; VAS)	18 months	Case series	(168)
M1	Neuropathic pain (2)	60 µs pulse width, 40 Hz, 2.5 V	100% (45% average; VAS)	40 months	Case series	(169)
	Neuropathic pain (6)	450 µs pulse width, 15 Hz, 5 V	16% (NR)	Up to 31 months	Case series	(162)
CMpf	Neuropathic pain (28)	NR	75% (>50; NR)	Median 14 months	Case series	(312)
Multi target						
CMpf and/or PAG/PVG	Neuropathic pain (3)	PAG/PVG: 60–110 µs pulse width, 10 Hz, 3.5–4.5 mA; CMpf: 60–90 µs pulse width, 128–132 Hz	100% (65.9% average; VAS)	36 months	Case series	(170)
	Neuropathic pain (3)	PAG/PVG: 90–120 µs pulse width, 5–10 Hz, 1–5 V; CMpf: 60–90 µs pulse width, 70–150 Hz, 2–2.5 V	NR (41% average; VAS)	Average 27 min	Case series	(313)
CMpf and/or VPL/VPM	Neuropathic pain (40)	210 µs pulse width, 130 Hz, 0.5–1.5 V (VPL/VPM) or 2.0–3.0 V (CMpf)	55.6% (>50% average; VAS)	48 months	Case series	(171)
PAG/PVG and/or VPL/VPM	Neuropathic pain (1)	NR	100% (60%; VAS)	8 months	Case series	(158)
	Neuropathic pain (7)	NR	57.1% (34% average; VAS)	6 months	Case series	(159, 160)
	Neuropathic pain (85)	200–450 µs pulse width, 5–50 Hz, 0.5–5 V	66.1% (50.3% average; VAS)	3 months	Case series	(172)
	Neuropathic pain (34)	PVG: 120–450 µs pulse width, 5–30 Hz, 0.8–4.5 V; VPL: 60–400 µs pulse width, 10–50 Hz, 0.7–4.4 V	76% (54% average; VAS)	Average 18.5 months	Case series	(173, 174)
	Neuropathic pain (15)	NR	NR (42% average; VAS)	Average 27 months	Case series	(173, 174)
	Neuropathic pain (36), nociceptive pain (10), NR (4)	NR	<50% (NR)	12 months	Multicenter open label	(175)
	Neuropathic pain (18)	60–450 µs pulse width, 5–50 Hz, 0.3–5.8 V	NR	Average 34 months	Case series	(176)
	Neuropathic pain (7)	NR	57.1% (34% average; VAS)	6 months	Single patient randomized controlled trial	(159, 160)
Multi target						
	Neuropathic pain (21)	60–250 µs pulse width, 25–120 Hz, up to 10 V	23.8% (>50%; VAS)	12 months	Case series	(177)
	Neuropathic pain (8)	5–35 Hz	PVG: 75% (NR), VPL: 50% (NR)	1 week	Case series	(178)
	Neuropathic pain (54), nociceptive pain (2)	PVG: 210 µs pulse width, 40–70 Hz; VPL: 210 µs pulse width, 60–90 Hz	39.2% (>50%; VAS)	Average 3.5 years	Case series	(179)
	Neuropathic pain (11)	100–500µs pulse width, 2–60 Hz, 0–10 V	100% (NR)	12–36 months	Case series	(180)

Articles were identified by reviewing two systematic review articles on DBS for chronic pain and removing duplicates (2, 181). ACC, anterior cingulate cortex; CMpf, centromedian parafascicular thalamic nucleus; M1, primary motor cortex; PAG, periaqueductal gray; PVG, periventricular gray; PLIC, posterior limb of the internal capsule; VPL, ventral posterolateral thalamus; VPM, ventral posteromedial thalamus; VS/ALIC, ventral striatum/anterior limb of internal capsule; NR, not reported; NRS, Numeric Rating Scale; VAS, Visual Analogue Scale. Available from the References (2, 126, 129, 132, 145, 148, 153–212).

**Table 2:** Stereotactic techniques used by DBS studies targeting chronic pain.

Region	Targeting location	Targeting hardware	Imaging	References
VPL	10–13 mm lateral to posterior commissure, macrostimulation 2 mm above to 5 mm below calculated target	Leksell frame	Stereotactic CT fused to preoperative MRI	(154)
	14 mm lateral to the intercommissural line at the level of the intercommissural plane and 10 mm posterior to the midcommissural point	Riechert-Mundinger frame and Zamorano-Dujovny semi-arc	Stereotactic CT fused to preoperative MRI	(171)
	10–13 mm posterior to the midcommissural point and between 5 mm below to 2 mm above it	Cosman-Roberts-Wells frame	Stereotactic CT fused to preoperative MRI	(172)
	6–8 mm posterior to the midcommissural point and 10–14 mm lateral, at the level of the anterior-posterior commissure line	NR	Stereotactic CT fused to preoperative MRI	(176)
	14–17 mm lateral to the midline at the level of the anterior-posterior commissure, 2–3 mm anterior to the posterior commissure	Leksell frame	Preoperative MRI	(177)
	10–13 mm posterior to the midcommissural point between 2 mm above and 5 mm below it, 14–18 mm lateral to midline,	Cosman-Roberts-Wells frame	Stereotactic CT fused to preoperative MRI	(178)
	12 mm lateral and 5–8 mm posterior to the midcommissural point at the level of the anterior-posterior commissure	Cosman-Roberts-Wells frame	Stereotactic CT fused to preoperative MRI	(160, 173)
	12–14 mm lateral and 0–2 mm anterior to the posterior commissure	Leksell frame	Stereotactic CT fused to preoperative MRI	(155)
	3–5 mm anterior to the posterior commissure, 0–2 mm above the intercommissural line, and 12–18 mm lateral to midline	Leksell frame	Preoperative MRI	(179)
	2–3 mm anterior to posterior commissure at the level of the anterior-posterior commissure, 14–17 mm lateral to midline	Leksell frame	Preoperative MRI	(210)
	11–15 mm lateral to the posterior commissure	NR	NR	(157)
VPM	12 mm lateral to intercommissural line at the level of the intercommissural plane and 10 mm posterior to midcommissural point	Riechert-Mundinger frame and Zamorano-Dujovny semi-arc	Stereotactic CT fused to preoperative MRI	(171)
	10–13 mm posterior to the midcommissural point and between 5 mm below to 2 mm above it	Cosman-Roberts-Wells frame	Stereotactic CT fused to preoperative MRI	(172)
	12 mm lateral to midline and 6–8 mm posterior to midcommissural point in the plane of the AC-PC line	Cosman-Roberts-Wells frame	Stereotactic CT fused to preoperative MRI	(159, 160)
	12–13 mm lateral to the midline at the level of the anterior-posterior commissure, 2–3 mm anterior to the posterior commissure	Leksell frame	Preoperative MRI	(177)
	3–5 mm anterior to the posterior commissure, 0–2 mm above the intercommissural line, and 10–12 mm lateral to midline	Leksell frame	Preoperative MRI	(179)
PAG/PVG	2–3 mm lateral to third ventricle at the level of the posterior commissure and 10 mm posterior to the midcommissural point	Cosman-Roberts-Wells frame	Stereotactic CT fused to preoperative MRI	(172)
	<10 mm below anterior-posterior commissure line, 3 mm lateral to lateral boundary of aqueduct and third ventricle	NR	Stereotactic CT fused to preoperative MRI	(176)
	<10 mm below the anterior-posterior commissure line, 5 mm lateral to lateral boundary of aqueduct and third ventricle	Cosman-Roberts-Wells frame	Stereotactic CT fused to preoperative MRI	(159, 160)
	2 mm lateral to the medial wall of the third ventricle at the level of the anterior-posterior commissure, 2–5 mm anterior to the posterior commissure	Leksell frame	Preoperative MRI	(177)
	10 mm posterior to the midcommissural point at the level of the anterior-posterior commissure, 3–4 mm lateral to the midline	Cosman-Roberts-Wells frame	Stereotactic CT fused to preoperative MRI	(178)
	2–3 mm lateral to the wall of the third ventricle, 2 mm anterior to the level of the posterior commissure, with most distal tip in the superior colliculus	Cosman-Roberts-Wells frame	Stereotactic CT fused to preoperative MRI	(173, 174)
	2–3 mm anterior to the posterior commissure, 2 mm lateral to the wall of the third ventricle, and 2 mm above and below the intercommissural line	Leksell frame with Zamorano-Dujovny semi-arc	Preoperative MRI	(179)
	8.2 mm posterior to the anterior commissure, 4.2 mm lateral to the midline, 1.1 mm superior to the level of the anterior-posterior commissure	NR	NR	(165)
CMpf	8 mm lateral to intercommissural line at the level of the intercommissural plane and 8 mm posterior to the midcommissural point	Riechert-Mundinger frame and Zamorano-Dujovny semi-arc	Stereotactic CT fused to preoperative MRI	(171)
	1.5 mm anterior to the posterior commissure at the level anterior-posterior commissure line, 1.5–2.5 mm lateral to the wall of the posterior third ventricle	NR	NR	(312)
VS/ALIC	3–5 mm anterior to junction of ALIC and anterior commissure	Leksell frame	Stereotactic CT fused to preoperative MRI	(163)
PLIC	16.7–24.4 mm lateral to the midline at the level of the anterior-posterior commissure, 4.5–5.9 mm posterior to the midcommissural point	Riechert-Mundinger frame	Stereotactic CT fused to preoperative MRI, DTI	(164)
ACC	20 mm posterior to the anterior tip of the frontal horns of the lateral ventricles	Cosman-Roberts-Wells frame	Stereotactic CT fused to preoperative MRI	(166, 167)
	20 mm posterior to the anterior tip of the frontal horns of the lateral ventricles	Maranello frame	Stereotactic CT fused to preoperative MRI	(168)
	20 mm posterior to the anterior margin of the lateral ventricles in the midsection of the gyrus	NR	NR	(165)
M1	Cortical region anterior to central sulcus with electrode in extradural space	NA	NA	(169)
	Paddle electrodes placed over the precentral gyrus in the epidural space	NA	NA	(210)

Only studies from Table 1 explicitly describing targeting techniques for specific brain regions were included. ACC, anterior cingulate cortex; CMpf, centromedian parafascicular thalamic nucleus; M1, primary motor cortex; PAG, periaqueductal gray; PVG, periventricular gray; PLIC, posterior limb of the internal capsule; VPL, ventral posterolateral thalamus; VPM, ventral posteromedial thalamus; VS/ALIC, ventral striatum/anterior limb of internal capsule; CT, computed tomography; MRI, magnetic resonance imaging; NA, not applicable. Available from the References (2, 126, 129, 132, 145, 148, 153–212).

**Table 3:** Summarized clinical effects at popular deep brain stimulation (DBS) targets.

	Average pain reduction			Responder rates^a^			
	Mean (%)	SD (%)	Studies (patients)	Mean (%)	SD (%)	Studies (patients)	Criteria
Single target							
VPL/VPM	60.8	12.4	2 (28)	78.3	16.7	3 (43)	>50–80%
PAG/PVG	62.5	-	1 (2)	87.5	17.7	2 (6)	
ACC	40.9	18.1	4 (49)	59.5	35.6	3 (42)	
VS/ALIC	-	-	-	11	-	1 (10)	>50%
M1	45	-	1 (2)	58	-	1 (8)	
Multi target							
CMpf and/or PVG	65.9	5.02	2 (6)	65.9	5.02	2 (6)	
PAG/PVG and/or VPL/VPM	45.7	10.8	6 (149)	64.9	26.9	8 (220)	

Single target posterior limb of the internal capsule (PLIC) and centromedian parafascicular thalamic nucleus (CMpf) are excluded. ACC, anterior cingulate cortex; M1, primary motor cortex; PAG, periaqueductal gray; PVG, periventricular gray; VPL, ventral posterolateral thalamus; VPM, ventral posteromedial thalamus; VS/ALIC, ventral striatum/anterior limb of internal capsule; SD, standard deviation.

^a^ Response thresholds are variably defined. Some studies use percent relief criteria, whereas others include response thresholds such as “satisfactory reduction in symptoms” or “decision to keep implanted pulse generator”.

### 2.1. Evidence for the involvement of specific brain regions in pain processing

A large body of predominantly correlative brain imaging literature in humans has been used to link activity in specific brain regions with different domains of pain processing (39, 43, 55–57). *In vivo* imaging of brain-wide blood oxygen level dependent (BOLD) signals with functional magnetic resonance imaging (fMRI) is the most widely used method for mapping brain activation during complex behaviors (43, 58, 59). The BOLD signal is known to reflect underlying local field potentials (LFP), which in turn correspond to stimulus-induced oscillations in the range of 30–150 Hz (60). In general, BOLD responses to painful experimental stimuli in Pain Matrix regions are reliably correlated with perceived pain intensity and consistently modulated by contextual factors that impact self-reported pain (42, 53, 54, 61–63). A subset of studies highlight that behavioral manipulations targeting specific (e.g., sensory, affective, and cognitive) domains of pain processing elicit predictable changes in associated brain regions (38, 64–66). For example, ACC activity is selectively reduced by hypnotic suggestion to reduce pain unpleasantness, consistent with the proposed role of ACC in the affective domain of pain perception (67). Studies of placebo and opioid analgesia suggest that frontal regions implicated in affective and cognitive processing domains, including the ACC, dlPFC, and OFC, are also well suited to modulate activity across the network, potentially through top-down recruitment of antinociceptive circuits in the PAG (40, 61, 63, 68–71).

Some argue that the evolution in terminology from “Neuromatrix” to “Pain Matrix” is inappropriate, as most included brain regions support domain-general, rather than pain-specific, processing (50). For example, salience-matched painful and nonpainful sensory stimuli elicit largely indistinguishable patterns of network activity, which challenges the specificity of observed BOLD activations to pain (72, 73). Moreover, many regions reliably implicated in pain processing are vulnerable to a reverse inference problem, in which a specific mental state (e.g., pain) is inferred from observed patterns of brain activation (e.g., thalamus, S1) (74, 75). Indeed, pain-relevant regions like the dorsal anterior cingulate cortex (dACC) and anterior insula (aIns) are the most commonly activated brain regions across the fMRI literature, regardless of the condition or process studied (76–78). For example, dACC is implicated in a range of specific functions ranging from attentional control and language processing to emotional expression and learning, which, although pain relevant, are not pain-specific (79, 80). In the context of pain, dACC activity is commonly cited as a proxy of pain aversiveness, yet a substantial number of studies have demonstrated that increased dACC activity is likely anti-nociceptive through top-down connections with the PAG (40, 68, 69, 81–86). Furthermore, most contemporary circuit models of pain omit regions where pain-related BOLD activations are often seen, such as the cerebellum, and where stimulation can profoundly alter pain perception, notably the primary motor cortex (M1) (87, 88). Thus, simple structure-to-function mapping is fraught with potential biases in interpretation, which challenge links between specific regional dysfunction and processing domains commonly disrupted in chronic pain (89, 90).

More recent advances in fMRI analysis and machine learning have enabled the development and characterization of the so-called “Neural Pain Signature” (NPS). The NPS is a weighted combination of multivariate patterned BOLD activity across pain-relevant brain regions that can accurately discriminate between painful heat and non-painful warmth or social rejection (91). The discriminative performance of the NPS offers proof of principle that activity across distributed networks is more closely linked to the subjective experience of pain than activity in individual regions (91, 92). The primacy of networks to pain processing is further supported by the observation that stimulation of individual brain regions rarely elicits pain and lesions to these regions seldom lead to specific changes in pain processing (50). Together, these observations have motivated a gradual transition away from focusing on regional dysfunction in specific structures and toward evaluating brain-wide network-level dysfunction across interconnected and functionally related brain regions.

### 2.2. Functional brain networks involved in chronic pain

Network analysis with resting state fMRI (rs-fMRI) has characterized a variety of intrinsic resting state networks (RSNs) defined by shared temporal fluctuations in spontaneous BOLD activity among interconnected brain regions. RSNs are believed to reflect the intrinsic functional architecture of underlying brain circuits (93) and correspond with established processing domains with relevance to pain perception (38, 50, 94). RSNs are typically estimated using normalized univariate Pearson correlation coefficients of the BOLD signal during periods of unconstrained (i.e., task-free) rest, known as resting state functional connectivity (rsFC) (95, 96). Functional networks estimated using rsFC correspond with structural networks defined by white matter pathways (97, 98) and with patterns of coactivation observed while performing relevant cognitive tasks (99–102). Further, the low frequency (<0.1 Hz) BOLD signals used to estimate rsFC have a similar spatial correlation structure to intracranial low frequency (<4 Hz) and γ-range (40–100 Hz) power (103, 104). BOLD correlations also predict the spread of intracranial evoked activations (105), illustrating their correspondence to underlying anatomical and physiologic network architecture. Importantly, RSNs are linked to the pathophysiology of a variety of neuropsychiatric disorders (10, 20) and modulation of specific RSNs with stimulation has been linked to improvements in corresponding symptom domains (106).

Several canonical RSNs have been directly implicated in the pathogenesis of pain, including a “salience network” (aIns, dACC, temporo-parietal junction, and dlPFC) thought to be involved in externally directed attention toward exogenous painful stimuli, a “default mode” network (DMN; posterior cingulate/precuneus, medial prefrontal cortex, and lateral parietal lobe) putatively involved in mind wandering away from pain, and a pain-specific descending modulatory network defined based on rsFC with PAG, among many others (100, 101, 107). A leading network model of chronic pain predicts that structural pathology within and between hubs of defined RSNs are associated with trait-level risk of developing chronic pain. Specifically, impairments in DMN organization and connectivity with the antinociceptive system are thought to enhance implicit attention to pain (94, 108). However, DMN pathology is not specific to chronic pain. Rather, DMN dysfunction is a commonly cited network correlate of a range of typically comorbid disorders characterized by rumination and introspection (109–111). Thus, just as individual brain regions, including the dACC, are implicated in divergent pain-relevant functions (e.g., enhancing pain aversiveness and recruiting antinociceptive circuits), DMN is routinely implicated in both pain-protective processes (e.g., distraction) and in ruminative and self-referential processes that are thought to increase the risk for developing chronic pain (110, 112–114).

### 2.3. Relevance of circuit models to chronic pain

Although contemporary circuit models of pain processing clearly suffer from a lack specificity to pain, more concerning for their translational potential to brain-based therapeutics is the consistent failure of models derived from studies of acute experimental pain to generalize to chronic pain. Most prior research has relied on studies of evoked brain activity during noxious stimulation in healthy volunteers, which may lack validity in the chronic pain state (43, 115). Whereas the NPS can predict acute experimental pain with >90% sensitivity and specificity, the same algorithm performs poorly when applied to natural fluctuations in chronic pain, which are more closely linked to patterns of rsFC observed during prolonged tonic pain (91, 116). Prior work directly comparing responses to acute experimental pain between healthy adults and chronic pain populations highlight consistent involvement of similar key brain regions (i.e., the “Pain Matrix”) in both acute and chronic pain, but more recent large-scale meta-analyses failed to identify any coherent patterns of regional dysfunction that reliably distinguish patients with chronic pain from healthy control subjects (117, 118). Other studies that examined spontaneous fluctuations of clinical pain suggest that recruitment of separate brain regions involved in reward and stimulus valuation (e.g., nucleus accumbens and medial prefrontal cortex) may be unique to the chronic pain state and even predict the likelihood of transitioning from subacute to chronic pain (119–123). Apkarian and colleagues have interpreted this pattern of central changes in chronic pain as evidence for two central hypotheses: that chronic pain is associated with (1) plastic reorganization of circuits that fundamentally alters the central processing of pain and enriches the sensory experience with emotional and cognitive links, and (2) a transition in the locus of pain from an external threat to a highly salient internalized disease state (115).

### 2.4. Summary

Theoretical accounts linking patterns of neural activity with the experience of pain conclude that understanding pain networks in terms of nociceptive processing alone fails to address how sensorimotor aspects of pain are enriched with cognitive and affective features in the chronic pain state (38, 43, 64, 94, 115). Models based on the “Pain Matrix” offer compelling interpretations for observed patterns of brain activity and their modulation by contextual variables (124). However, these models fall short when translating insights from acute experimental pain to circuit-level dysfunction in chronic pain. With respect to analgesic brain stimulation, these models suggest several testable hypotheses about which brain regions should be targeted and through which domains brain stimulation may be effective. Combining network analysis with targeted brain stimulation has great potential to enrich correlative human brain imaging data with causal evidence (8, 14, 125). In subsequent sections, we integrate prior experience with brain stimulation for pain with the region and network-level hypotheses summarized below. Because stimulation can variably inhibit or excite brain activity, and because mechanisms underlying such tissue effects are poorly understood, we offer hypotheses for both phenomena (126, 127).

### 2.5. Testable Hypotheses

I. If brain stimulation inhibits local activity and creates a virtual lesion (127), then stimulation of loci within defined pain transmission circuits (e.g., thalamus and somatosensory cortex) should inhibit pain.
II. Excitatory brain stimulation that activates brain loci associated with pain suppression, especially opioidergic PAG and regions with known connections to PAG (41), should inhibit pain.
III. Stimulation of loci from circuits linked to functionally distinct processing domains should have separable effects on the sensory, emotional, and cognitive aspects of pain.
IV. Network dysfunction models of chronic pain (13, 38, 94, 115, 128) predict that stimulation at various loci within a network should elicit similar pain outcomes.

## 3. Prior brain stimulation for chronic pain identifies multiple viable targets and mechanisms for pain relief

Various neuromodulation strategies are described for brain-based treatment of neuropsychiatric disorders and chronic pain. Deep brain stimulation (DBS) involves neurosurgical implantation of intracranial electrodes for direct electrical stimulation of specific brain regions and is currently FDA approved to treat movement disorders, epilepsy, and obsessive-compulsive disorder (OCD, through humanitarian exemption), with additional evidence of benefit in major depressive disorder (MDD) and chronic pain (129–132). While fundamental mechanisms of action of DBS for pain are not clearly defined, it is postulated that stimulation could modulate neural activity via a temporary lesion effect or through direct excitation of surrounding structures (126, 127). Transcranial magnetic stimulation (TMS) is a more recently developed non-invasive neuromodulation technique that utilizes magnetic fields to induce electrical currents beneath the skull (133). TMS can excite or inhibit superficial cortical structures depending on the frequency of stimulation (134–136). High frequency (i.e., >5 Hz) excitatory repetitive TMS (rTMS) is currently FDA-approved as a treatment for MDD at left dlPFC (137) and for OCD at dorsomedial PFC/ACC (138). There is accumulating evidence that high frequency rTMS at primary motor cortex (M1) can be effective for chronic neuropathic pain in select patients (6, 7, 139). MRI-guided low-intensity focused ultrasound (MRgFUS or LI-FUS) is an emerging form of non-invasive neuromodulation that is capable of precisely modulating brain activity in deep subcortical regions that are out of reach of conventional TMS probes (4, 5, 140, 141). Additional stimulation modalities such as transcranial direct current stimulation (tDCS) are described for chronic pain but are excluded from the present targeting discussion considering their more limited spatial precision.

### 3.1. Periaqueductal gray (PAG) and periventricular gray (PVG)

PAG is a midbrain nucleus that extends along the cerebral aqueduct from the locus coeruleus to the posterior commissure. PVG is the diencephalic extension of PAG rostral to the posterior commissure. Whereas PVG was the putative stimulation target in most early DBS trials, most preclinical antinociception literature focuses on PAG. Limited post-mortem evaluations found that similar analgesic responses could be obtained from stimulation in both regions, even within a single patient, and that final electrode locations often differed slightly from planned trajectories (i.e., PVG directed contacts within PAG, and vice versa) (142). We therefore combine our discussion of both regions in this section. PAG was the first brain region to be implicated in endogenous pain modulation and has subsequently been linked to additional homeostatic functions including autonomic control, aversive learning, and coordination of defensive escape/avoidance behaviors (143–151). A substantial body of preclinical evidence convincingly shows that PAG stimulation is antinociceptive (41, 83, 143, 144, 149, 152). Small case series of PAG/PVG DBS for chronic pain reported variable response rates, which may reflect the broad range of stereotactic hardware, targeting coordinates, stimulation parameters, and follow-up times in prior studies (Tables 1, 2). Stereotactic implantation of DBS electrodes is typically performed contralateral to pain, targeting the level of the superior colliculus 2–3 mm lateral to the third ventricle, corresponding with the expected location of the PVG (Table 2). In most prior studies, final electrode position is informed by intraoperative awake macrostimulation using a range of parameters titrated to analgesic effect (172, 178, 179). Subjectively, stimulation of the PAG/PVG elicits a warm sensation that displaces pain in the affected area, a response that has been used to guide final electrode position (160, 180). Other studies have used objective neurological signs such as head bobbing or ocular deviation for target determination (173, 177). Prior studies that reported frequency-dependent effects during macrostimulation found that lower frequency stimulation between 5 and 50 Hz elicited analgesia or pleasant paresthesia, whereas larger stimulation amplitudes and higher frequencies >50 Hz were associated with adverse experiences such as hyperalgesia or anxiety (Figure 1B) (177, 179). Although preclinical work strongly suggests that PAG-mediated analgesia is opioid dependent, clinical experience with PAG/PVG DBS suggests that stimulation may produce analgesia through both opioidergic and non-opioidergic mechanisms (186, 213, 214). Notably, PAG/PVG stimulation has also been reported to improve neuropsychiatric outcomes like mood, anxiety, and quality of life (176). Despite the use of intraoperative macrostimulation to elicit favorable prognostic symptoms and signs (which may account for structure-function variability across patients), stimulation of PAG/PVG nonetheless elicits highly variable and often incomplete analgesia (Table 3). The dissociable pain relieving and anxiogenic effects of low and high frequency acute stimulation are suggestive of distinct activation of antinociceptive vs escape/avoidance circuitry at different frequencies. To our knowledge, this has not been shown in humans. There may be alternative explanations (e.g., variability of local neuronal populations along the dorsoventral and rostrocaudal PAG/PVG axes) that are difficult to evaluate without the benefit of precise coordinates or detailed post-operative imaging from prior studies [but see (142)]. Taken together, the frequency dependence of analgesic PAG/PVG stimulation supports Hypothesis II, which together with widespread effects on sensory, affective, and cognitive pain processing domains suggests that PAG stimulation either enhances pain suppression or exerts widespread network effects.

### 3.2. Sensory thalamus

The sensory thalamus is composed of the ventral posterior lateral nucleus (VPL) and ventral posterior medial nucleus (VPM), which receive somatotopically organized afferent fibers from the spinothalamic tract and project to primary somatosensory cortex, as well as cognitive and limbic association areas (190). The central location of thalamus within pain transmission circuits made it an attractive early candidate for neuromodulation. Thalamic DBS emerged as one of the first viable applications for neuropathic pain in the 1970s but ultimately fell out of favor after two multicenter trials failed to meet efficacy criteria and industry partners largely abandoned their pursuit of FDA approval (189). These trials were subsequently criticized for the inclusion of heterogeneous chronic pain etiologies and lack of appropriate follow-up, which may have contributed to observed inefficacy (3). DBS target determination for thalamic stimulation takes advantage of the known mediodorsal somatotopic organization of the ventroposterior thalamus, such that VPM as the target for facial pain and the VPL for body or limb pain (154). Anatomically, VPM trajectories are typically 2 mm towards the midline and anterior to the posterior commissure relative to the VPL trajectory (Table 2). Several case series report acute analgesic effects from thalamic stimulation when pain is supplanted with paresthesia upon stimulation (Figure 1B). As with prior PAG/PVG DBS studies, this finding is often used to inform the final electrode position (153, 154, 158). However, despite the use of macrostimulation to ensure that stimulation effects localize to the painful anatomical region, personalized targeting alone is insufficient to ensure adequate analgesia (172, 179). Thalamic stimulation is generally ineffective for thalamic and central post-stroke pains, and across pain disorders, response rates and pain relief estimates are comparable to PAG/PVG DBS (Table 3). Adverse effects of stimulation ranged from reports of increased pain to dystonic movements caused by internal capsular stimulation (172, 179). The variability of outcomes with thalamic DBS suggests that stimulating pain transmission circuits, even when confirmed by positive sensory phenomena in the affected region, is insufficient to reliably modulate the overall experience of chronic pain, especially for neuropathic pain resulting from central (i.e., brain) injury (Hypothesis I).

### 3.3. Anterior cingulate cortex (ACC)

ACC is a heterogeneous midline cortical structure implicated in a range of emotional, cognitive, and motivational functions (79, 215). ACC exhibits a gradient of rsFC along its rostrocaudal axis ranging from affective and evaluative regions like OFC and amygdala anteriorly to action planning regions like frontal eye fields (FEF) and premotor cortex posteriorly (201, 202, 206). ACC DBS extends from prior experience with bilateral stereotactic cingulotomy for refractory pain, which was found to elicit profound and specific improvements in the affective and motivational aspects of chronic pain (216). ACC has more recently gained favor as a means of targeting the burden of suffering largely driven by the affective domain of chronic pain (2, 217). Spooner reported the first case of bilateral ACC DBS targeting an area 20 mm posterior to the anterior tip of the lateral ventricles in the midsection of the gyrus and reported slight improvements in pain visual analog scores (VAS) compared with PAG stimulation in the same patient (165). Intra-operative stimulation at 130 Hz did not yield any apparent sensory phenomena or pain relief (Figure 1B). Since then, most case series stimulating the ACC for neuropathic pain have applied high frequency (130 Hz) stimulation, noting variable initial responses and generalized loss of efficacy at longer follow-up times. Some of these studies have targeted the cingulum white matter bundle instead of overlying cortex (168). Patients receiving ACC DBS across multiple studies describe their pain as less emotionally unpleasant even if sensory/discriminative judgements about pain remain intact (unpublished personal communication to PS). Accordingly, ACC stimulation has been associated with significant improvements in several measures of quality of life and social functioning (167, 168). These findings support the hypothesis that disparate brain circuits may be involved in processing different domains of pain processing (Hypothesis III). Compared with more spatially restricted anatomical structures like PAG and Thalamus, ACC is a much larger and more structurally and functionally heterogeneous region, which may account for the slightly lower response rates and pain reduction effect sizes seen in prior studies (Table 3).

### 3.4. Ventral striatum (VS)/internal capsule (IC)

DBS of the ventral striatum and anterior limb of the internal capsule was motivated by prior work linking VS with the affective domain of pain processing and by the proven efficacy of VS/IC DBS for other comorbid neuropsychiatric conditions such as OCD and MDD (196, 198, 205). VS dysfunction and connectivity with prefrontal cortex is an established biomarker of pain chronification and is therefore a particularly attractive candidate stimulation location for patients with chronic pain (121–123, 218). However, a recent double-blind randomized sham-controlled trial targeting these regions did not meet its primary endpoint of greater than 50% pain intensity reduction in more than 50% of patients. As with prior ACC DBS, targeting VS/IC did show significant improvement in several outcome measures quantifying the affective dimension of pain (Figure 1B) (163). Results of ACC and VS DBS studies suggest that convergent effects on pain affect can result from stimulating separate areas of a subnetwork involved in affective processing (Hypothesis IV). However, it remains unclear whether patients that respond to stimulation at one site within a critical network would exhibit similar outcomes at the other site within that network, and whether stimulation of both regions simultaneously would have synergistic effects.

### 3.5. Primary motor cortex (M1)

Motor cortex stimulation (MCS) first emerged as a viable target for chronic pain after the serendipitous discovery that M1 stimulation reduced bursting activity in the ischemic penumbra of a feline thalamic stroke model (219). Despite early clinical success of MCS, pain outcomes are similarly unreliable as with other DBS targets (220–222). In the 1990s, TMS began to reproduce and predict potential benefits of MCS with non-invasive stimulation of M1 contralateral to pain (223–225). M1 continues to be among the most effective invasive and non-invasive stimulation targets for pain (6, 7, 139, 226), but is not yet FDA approved for this indication in the US. Although the exact mechanisms of pain relief from M1 stimulation are unknown, several hypotheses are proposed (88, 220). High-frequency excitatory M1 rTMS combined with brain imaging suggests that M1 stimulation elicits activity in a distributed network of brain regions implicated in pain processing, including: thalamus, PAG, insula, ACC, and the medial PFC region implicated in pain chronification (122, 227, 228). MCS excites PAG in preclinical models (229) and elicits endogenous opioid release in ACC and PAG in humans that is in turn correlated with pain relief (230). Analgesia from M1 TMS, like PAG/PVG DBS (in certain cases), is naloxone reversible (231, 232). There is some evidence that M1 stimulation is best for pain involving the contralateral face and arm and performs less well for lower extremity and widespread pain syndromes, suggesting somatotopic effects (233, 234). Overall, despite its omission from most circuitry models of chronic pain, M1 stimulation activates a network of pain-relevant brain regions that predict clinical improvement in pain scores. Most recently, this same region was shown to be a common network hub linking brain lesions that cause pain, suggesting that M1 may be a network target with diffuse influence over other implicated brain regions (128). Although this is an attractive hypothesis, the limited benefit of M1 for widespread and lower extremity pains argues for a more specific somatotopic mechanism.

### 3.6. Other targets

A variety of cortical regions have been explored as targets for chronic pain using high frequency rTMS. The left dorsolateral prefrontal cortex (dlPFC) is a neocortical structure involved in higher order cognitive and executive functions and component of the salience RSN. It is an established FDA-approved rTMS target for refractory major depressive disorder (MDD) and has been studied for a range of pain conditions (7, 137). Its direct role in pain processing is unclear, but activity in this region has been linked to selective attention to nociceptive stimuli (42). Meta-analyses of left dlPFC rTMS suggest no reliable benefit for chronic pain, although there is some evidence that left dlPFC may be effective for certain widespread pain conditions like fibromyalgia, mTBI headache, and pain with comorbid MDD (6, 139, 208, 235–237). One recent study evaluated rTMS at the dACC and posterior Insula (pIns) regions implicated in sensory and affective pain processing using a double-cone TMS coil design capable of stimulating deeper cortical structures (238). Although dACC stimulation reduced self-reported anxiety and pIns stimulation modulated experimental pain detection thresholds, neither target was clinically efficacious for chronic pain. This pattern of results offers further support for the assertion that regions implicated in nociceptive processing may not be viable analgesic targets in chronic pain and that modulation of emotional processing alone may not meaningfully improve the experience of pain. However, it is also possible that the relative loss of precision with rTMS at deeper targets may be contributing to the lack of clinical efficacy.

### 3.7. Multi-target stimulation

If the experience of pain relies on parallel processing in distributed brain networks, multidimensional pain relief may require simultaneous targeting of separate subnetworks. Motivated by earlier findings that thalamic DBS outcomes were better for patients with deafferentation pains (e.g., rhizotomy, spinal cord injury) and PAG/PVG DBS outcomes were better for nociceptive pain (e.g., cancer), Hosobuchi and colleagues hypothesized that simultaneous stimulation of both regions would lead to more comprehensive pain improvements (180). All eleven patients in the cohort reported satisfactory pain relief and continued to use DBS as their primary therapeutic modality at longer follow ups. Although limited by the absence of blinding and control stimulation, these findings suggest that simultaneous targeting of pain transmission and modulation pathways may be more effective than single-target stimulation. Combined acute stimulation of the PAG/PVG and the centromedian parafascicular (CMpf) nucleus of the thalamus, a thalamic nucleus implicated in attention and arousal, was show to relieve pain by an average of 41% within 30 min that lasted 2 h (209). A follow-up study by Hollingworth et al. investigated dual frequency DBS of the PAG/PVG and CMpf, demonstrating 100% response rate with 65.9% average VAS pain intensity reduction in three patients with medically refractory chronic pain at 3 years post implantation (Table 3) (170). While these results have yet to be replicated in larger sample sizes with blinded, randomized, and controlled study designs, they imply that simultaneously targeting regions involved in distinct aspects of pain processing may be more efficacious that single site DBS.

### 3.8. Summary

Despite the anatomical precision afforded by pain's somatotopic representation, stimulation of targets along defined transmission, central processing, and descending modulation pathways have failed to yield consistent relief across patients. Importantly, although prior case series of DBS have personalized electrode targets for individual patients using objective signs and subjective sensory reports during intra-operative macrostimulation, outcomes across all defined targets remain modest and unreliable (Table 3). Thus, although nociceptive stimuli may enter awareness through defined pathways, the multidimensional experience of pain that drives the burden of suffering may be difficult to capture by stimulating individual regions of interest to pain processing. Our review of prior brain stimulation highlights that while stimulation of individual regions rarely leads to complete relief, different domains of pain processing can be selectively manipulated by stimulating specific regions (e.g., affective domain improvements with ACC and VS stimulation). Importantly, emerging multi-target stimulation data suggest that stimulation of hubs from distinct networks may overcome some of the limitations of stimulating individual brain regions (13, 170, 180). However, even multi-target stimulation of popular brain regions only works for a minority of patients. Overall, prior experience with DBS and TMS for chronic pain supports two key observations: (1) no single brain region is universally efficacious for pain relief, yet (2) convergent relief can be obtained at a variety of stimulation locations. Taken together, these results highlight that a personalized network-informed approach to stimulation that seeks to simultaneously modulate multiple regions or subnetworks involved in distinct aspects of pain processing may lead to more reliable effects on the multi-dimensional experience of pain.

## 4. Network informed brain stimulation for neuropsychiatric disorders

Before turning to new targeting methods, it is first useful to review established fMRI-guided brain stimulation methods. The gradual development of rTMS targets for major depressive disorder (MDD), beginning with a single dysfunctional left dlPFC region and culminating in the development of rsFC-informed targets, highlights that network-informed stimulation can improve clinical outcomes. The selection of a consensus depression-relevant subgenual ACC (sgACC) region with which to explore rsFC with candidate rTMS targets was ultimately the key first step toward viable personalized neuromodulation for MDD (239). Although PAG and sensorimotor cortex are proposed as viable consensus targets for chronic pain (13, 128), rsFC with these regions has not yet been linked to improved stimulation outcomes. Below we discuss the strengths and weaknesses of existing targeting methods in MDD before proposing a novel approach that replaces *a priori* defined regions of interest with regions informed by individual network topologies.

### 4.1. TMS for depression supports network-informed targeting

The FDA-approved left dlPFC target for MDD was initially motivated by prior observations that left dlPFC lesions increase the risk of depression and that metabolism in this region increases with successful antidepressant treatment (136, 240, 241). Variable outcomes across early rTMS studies began to highlight that heterogeneity in clinical response was at least partially driven by variability in the underlying left dlPFC anatomical target, which is among the most structurally and functionally variable brain regions across individuals (27, 29, 242, 243). Subsequent studies sought to improve the consistency of stimulation across patients by targeting a region 5 cm anterior to the location where stimulation elicited a reliable finger twitch (137). Variability in head size motivated yet more precise measurements, guided by the 10–20 electroencephalogram (EEG) montage, to identify a patient-specific left frontal F3 point (244). Although the “Beam-F3 approach” has not been shown to be more clinically efficacious than the 5 cm point in head-to-head trials, it more consistently engages the left dlPFC and is therefore the method currently endorsed for clinical use in MDD by the Clinical TMS society (8, 245). More recent attempts to transition from coarse scalp landmarks to individual coordinates derived from group-level comparisons between MDD and healthy controls have unfortunately not led to any substantive improvement in clinical outcomes (8).

Variability in the specific location of the left dlPFC stimulation target across prior studies ultimately proved fruitful for subsequent mechanistic studies seeking to characterize network parameters associated with favorable clinical outcomes (14, 17, 106, 239, 246). Across prior rTMS trials, rsFC between the stimulated left dlPFC target and a region of sgACC routinely implicated in depression was reliably correlated with improvement in symptoms (125, 247, 248). This key finding motivated more recent attempts to use rsFC with sgACC to prospectively identify the “best” dlPFC region to stimulate for depression (15, 17). However, there are notable caveats to this approach. First, prior studies have compared rsFC estimates within a restricted dlPFC search space. It is therefore unclear whether the dlPFC target identified using rsFC with sgACC is the best treatment location for MDD, or whether there may be alternative brain regions outside of dlPFC where stimulation is more efficacious. Second, correlations between clinical outcomes and rsFC in most prior studies were generated using a large normative sample of healthy adults without depression, from a standardized open-source “connectome” dataset (58, 239, 249). In other words, although coordinates used to generate rsFC estimates were derived from individual patients, the resulting rsFC parameter for each set of coordinates was estimated using a separate large, unaffected sample group.

The use of large normative samples to explore network properties of neuromodulation targets is motivated by limitations of the fMRI BOLD signal, which is characterized by low signal-to-noise ratio (SNR), poor signal coverage in a variety of structures of interest (especially orbitofrontal cortex and inferior temporal lobes), and low temporal resolution (1.5–2 s in most studies), which together pose significant barriers to generating reliable and valid estimates for individual patients (58, 59). Averaging across large samples improves statistical power, which is linked to more stable parameter estimates but assumes homogeneous structure-function relationships across patients (250–252). Using large normative datasets to generate rsFC estimates has dramatically advanced our understanding of the network-level correlates of behavior and yielded powerful new methods to understand a variety of neuropsychiatric conditions that may be better understood as “circuitopathies” (241, 249, 253, 254). However, the same group that first identified anticorrelation with sgACC as a predictor of left dlPFC rTMS efficacy also showed that the estimated dlPFC target derived from individual subject data diverged from the group average prediction (255). Although subsequent studies testing the generalizability of estimates derived from the normative connectome highlight that datasets from affected clinical populations yield similar estimates (17, 106, 128, 254) and that the direction and magnitude of sgACC rsFC estimates derived from individual subject data are similar to population estimates (15), it remains unclear whether population level estimates of network organization will translate to prospective target selection for individual patients.

### 4.2. Precision neuroscience may inform personalized stimulation

Although single-subject fMRI applications have been fraught with potential methodological confounds, more recent technical developments in MRI acquisition and preprocessing have improved both the SNR and temporal resolution of acquired fMRI data. Specifically, multi-band fMRI acquisition protocols, and more recently, multi-echo fMRI (among other preprocessing advancements) have been shown to improve the reliability of single subject network estimates (24, 256). Serial imaging studies of highly sampled individuals suggest that approximately 10 min of data are needed to estimate reliable networks in individual patients using these methods (256), compared with approximately 45 min using standard acquisition techniques (23, 257). These emerging single-subject applications, often termed precision fMRI (pfMRI), provide a viable counterargument to recent high-profile indictments against the inferential power of population-level fMRI analyses, which suggest that thousands of patients are required to identify reliable brain behavior relationships (23, 252, 258). Together with pervasive heterogeneity in preprocessing and deficient statistical methods across the fMRI literature (259, 260), such power concerns are thought to be contributing to a widespread generalizability crisis in neuroscience (261). However, it seems equally likely that noisy and unreliable estimates at the group level are a direct consequence of averaging across important individual differences in brain organization (23, 262, 263). Indeed, brain networks are highly variable from person to person (26–28, 243), but generally consistent within individuals (22, 24, 25, 30, 31). Recent work combining individualized network estimates with MRI-guided dlPFC rTMS underscores that targeting the same anatomical area engages different functional regions and RSNs in different patients, providing further support for the assertion that individual estimates are more likely to yield reliable targeting estimates for neuromodulation (19, 23). The critical importance of personalized targeting finds additional support in the cognitive neuroscience literature, where targeting TMS with individual subject fMRI data leads to significantly greater effect sizes across a range of behavioral experiments compared with anatomical, group-level functional, and scalp landmark based targeting approaches (264).

### 4.3. Applications of precision neuromodulation

There are few clinical targeting implementations that validate group-level estimates with individual subject data (11, 15) and fewer still that have used baseline rsFC to guide prospective targeting for individual patients (9, 16, 19). To our knowledge, only one group has successfully used individual subject fMRI data to prospectively generate a targeting prediction for subsequent TMS (9, 16). Although emerging data from the Stanford Neuromodulation Therapy (SNT) protocol suggests that personalized targeting estimates may improve outcomes for patients with MDD, the authors incorporate two parallel advances to conventional TMS protocols that limit attribution of benefit to personalized targeting. Patients receiving SNT receive a novel and higher dose intermittent theta burst TMS protocol that alone may explain the impressive remission rates, greater than 75%, seen in the study. This approach was compared with sham stimulation, but not active stimulation of a control target, leaving considerable uncertainty regarding the specific contribution of personalized targeting to the overall result. Moreover, although the SNT method uses individual patient fMRI data to generate targeting estimates, the search space for the personalized target is constrained by both the key sgACC region and planned dlPFC stimulation region, such that the targeting algorithm refines dlPFC placement to optimize sgACC connectivity but does not allow for identification of alternative targets outside of the established dlPFC search space. Thus, while the method offers compelling evidence that precision is key to optimize targeting, there are currently no reliable methods to identify optimal sites from a brain-wide list of potential candidates.

### 4.4. Summary, caveats, and emerging applications to chronic pain

Experience with hypothesis-driven target selection in MDD suggests that rsFC with a single region of *a priori* interest (i.e., sgACC) can explain a substantial amount of variability in treatment outcomes. Prior work suggests that using a single depression-relevant sgACC region to generate rsFC estimates may also be a viable means to generate prospective, rsFC-based targeting estimates for individual patients. Results linking rsFC between efficacious TMS and DBS targets across neuropsychiatric disorders suggests that identifying a network of relief (i.e., rsFC patterns that link all effective sites across stimulation modalities and exclude ineffective ones) should facilitate more consistent stimulation outcomes for patients with a variety of disorders (13). However, this model would predict that sgACC should be an effective DBS target, yet only 4 of 6 patients obtained benefit in the first sgACC DBS study (265). Moreover, a larger multi-center sgACC DBS trial was halted for underperformance relative to the preceding open label trial. Remission rates for the sgACC DBS target in subsequent open label reports were around 30% (266, 267). Even more difficult to reconcile are the markedly higher remission rates seen in the SNT rTMS trial that used sgACC to identify a personalized left dlPFC target. As above, results from SNT may reflect the higher “dose” of electrical stimulation inherent to the theta-burst protocol, or unique stimulation parameters of rTMS relative to DBS, but may also reflect the specific rsFC method used to derive the dlPFC target. Rather than using rsFC with the consensus sgACC region of interest, a hierarchical agglomerative clustering algorithm was used to identify unique functional sgACC subunits based on patterns of brain-wide connectivity. Next, optimal dlPFC connectivity with each subunit was used to inform the final rTMS target. Such an approach may overcome structure-function limitations inherent to using a single group-average sgACC region for all patients. It is also possible that whole-brain agglomerative clustering may be unintentionally identifying dlPFC nodes based on as-yet unknown global network properties that have little to do with specific connections between sgACC and dlPFC.

In chronic pain, PAG and sensorimotor cortex (M1/S1) are proposed as viable candidate network hubs for rsFC-based targeting, akin to the established sgACC region in MDD (13, 128). However, as reviewed in Section 3, stimulation at both PAG and M1 elicits variable analgesia (Table 3). Although prior work predicts that M1 rTMS should be more efficacious than left dlPFC rTMS for patients with chronic pain expressly because M1, but not dlPFC, is significantly anticorrelated with PAG in the normative connectome dataset, there are reports of clinical pain benefit from left dlPFC rTMS for individual patients and dozens of reports of rTMS at M1 showing modest pain relief and relatively low responder rates, especially for widespread and lower extremity pain conditions (6, 7, 139). Incidentally, the M1 stimulation target with best evidence for clinical efficacy across pain conditions already overcomes several targeting limitations posed by other neuropsychiatric disorders. Targeting M1 requires elicitation of an observable motor response in the affected (contralateral) extremity for each patient, similar to personalization with intraoperative macrostimulation in PAG and Thalamic DBS [see (6, 139, 220) for review]. M1 stimulation reliably engages a distributed network of pain-relevant regions (227, 228, 230) and has recently been shown to modulate between network connectivity between the DMN and salience networks (268), highlighting multiple viable network mechanisms for analgesia. Moreover, interindividual variability in representations of acute pain are greatest in midline prefrontal regions commonly implicated in the transition to chronic pain (32, 34) and lowest in regions like M1 (29). Despite these advantages, response rates for M1 TMS are highly variable, modest, and often short-lived. The lack of viable network informed targets for chronic pain and failure of even the best contemporary targeting models in other disorders for a substantial subset of patients highlights the clear need for alternative strategies to identify more robust and generalizable network-informed imaging biomarkers for brain stimulation (36).

We propose that our emerging understanding of the profound interindividual variability in functional networks demands a personalized approach that uses individual subject fMRI data to generate predictions of individually optimized stimulation targets (23, 269, 270). The dearth of reliable neural correlates of chronic pain in clinical populations and the observation that both intrinsic RSNs and patterns of evoked activity to pain are profoundly variable across individuals suggest that any attempt to identify a single optimal brain region for analgesic stimulation will fail for a substantial number of patients. Although it is attractive to consider the possibility that a small number of viable targets may be generalizable across patients, seeking to manipulate single brain regions or even single networks may be overly reductive, as this approach largely ignores the substantial contribution of dynamic interactions between brain regions to perception and behavior (99, 100). Considering the apparent limitations of selecting brain regions based on rsFC with a subset of defined anatomical regions of interest, we hypothesize that a truly network-informed approach to stimulation should instead consider the overall topology of the network to identify personalized regions where stimulation is most likely to have a widespread impact on pain-relevant regions and subnetworks. This approach fundamentally reframes the characteristics of an “ideal target” away from specific neuroanatomical regions defined at the population level and toward estimates that carry more information about the overall structure and organization of individual brains (i.e., topology) to characterize the functional role of each candidate region within the overall network. Below we elaborate on this approach and review novel network analysis methods that may be more robust to variability in network topology across individuals.

## 5. Modeling network dysfunction in pain to inform biomarker development

**Figure 2:**
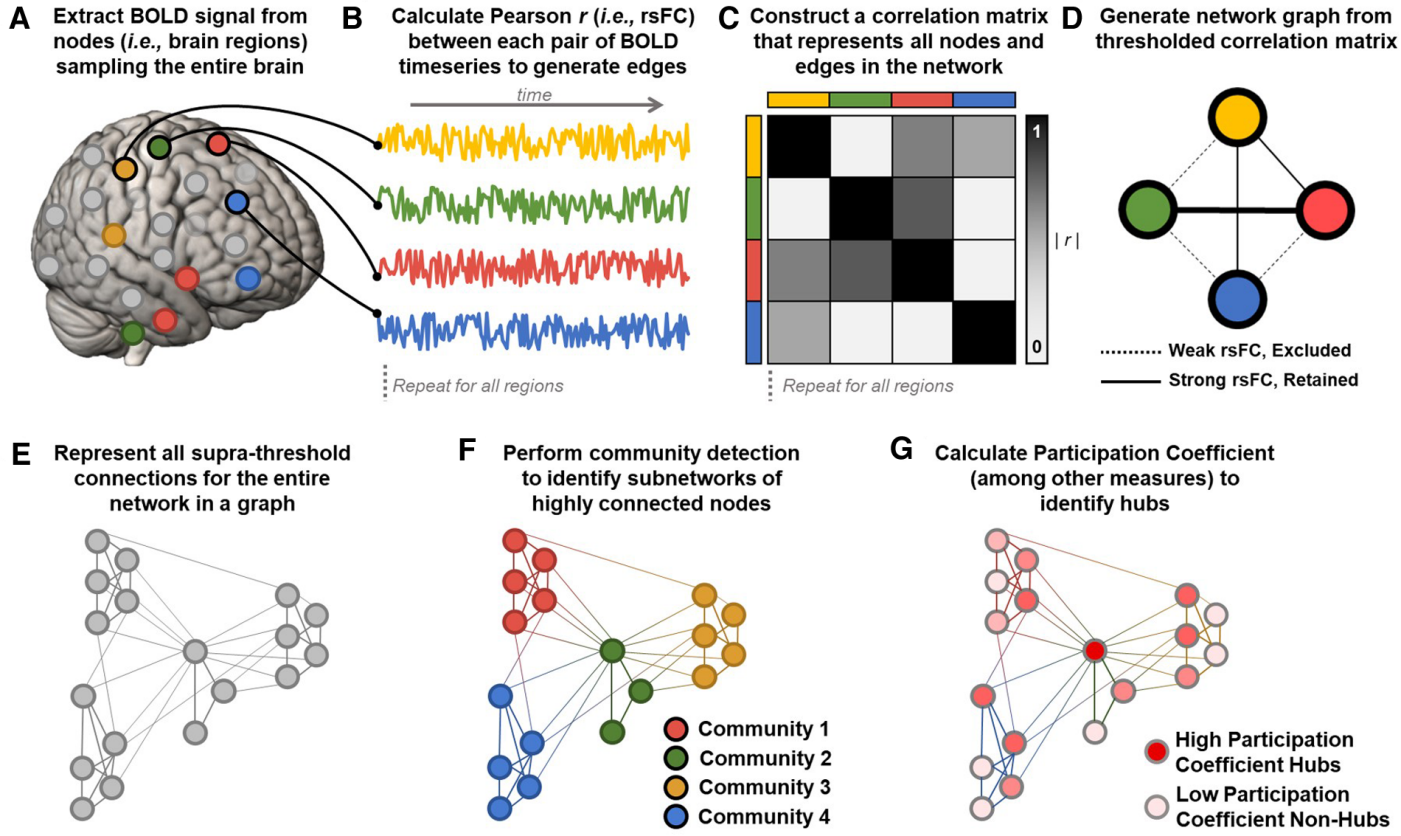
Calculating graphs from fMRI data to estimate network parameters. (**A**) Resting state blood-oxygen-level-dependent (BOLD) signals are extracted from a parcellation of *nodes* sampling the entire brain. (**B**) Representative BOLD timeseries from each node are used to calculate Pearson correlation coefficients (*r*) between each pair of *nodes* to generate *edges* reflecting resting state functional connectivity (rsFC) between regions. (**C**) Z-transformed rsFC between all possible pairs of regions is represented as a correlation matrix. (**D**) The most significant functional connections in the graph (typically the top 2%–10% of rsFC values) are used to construct a network graph consisting of above threshold *nodes* and *edges*. (**E**) An example of an entire brain network topology represented as a graph, generated using the methods outlined in **A–D**. (**F**) Community detection algorithms (e.g., Infomap, Louvain) assign each node to a community (i.e., *module*) consisting of color-coded groups of nodes that are more strongly connected with each other than with nodes in other communities. Network *modularity* can be calculated by comparing within- and between-module connections. In this example, four communities are detected, with colors corresponding to the four pain processing subnetworks shown in Figure 1A. Note that community detection algorithms are data-driven and need not correspond pre-defined sensory, affective, cognitive, and regulatory subnetworks. (**G**) Once nodes are assigned to communities, additional parameters like *participation coefficient* can be calculated to identify nodes connected to many different communities. In this example, a central node from the pain modulatory subnetwork (green in panel **F**) is maximally connected to all four communities and is an ideal candidate connector or integrator hub (dark red). Peripheral nodes in each subnetwork are least hub-like (light pink).

An emerging network neuroscience literature using mathematics from graph theory has established powerful methods to characterize integrated local and global network organization unconstrained by the specific anatomical location of the component parts of the system. A variety of complex systems—ranging from transportation and telecommunications infrastructure to metabolic and neural networks—are intuitively represented and analyzed using mathematics from graph theory (271–276). Graphs are constructed from *nodes* consisting of individual units in the system (e.g., airports, neurons) and *edges* consisting of connections between units (e.g., flights, axons). The key advance of graph theory over conventional approaches to estimating rsFC (i.e., with RSNs) is representing brain regions as *nodes* and rsFC between pairs of regions as *edges* in a graph in order to facilitate the computation of a variety of neurobiologically meaningful summary estimates that simultaneously capture properties of individual regions and their relationship to the overall network topology (277). To generate a network graph, rsFC between all pairs of nodes (i.e., regions) is used to construct a correlation matrix, which is then normalized, thresholded, and binarized to construct a network graph that visually represents the entire system (Figures 2A–E). A variety of widely available open-source toolkits are then used to estimate region and network level parameters from the network graph (271, 277, 278). Notably, graph theory can be used to represent a broad range of related network properties, including structural connections estimated from white matter integrity between regions, functional connections estimate with rsFC between regions, and regional transcriptomes, among other emerging applications (271). Here, we primarily focus on functional networks defined with rsFC for consistency. A comprehensive summary of the graph terms relevant for network neuroscience is beyond the scope of the current review, but fall into measures of integration, segregation, and centrality. We suggest that the reader review (277) for a detailed description of network terms and their functional significance and (279) for a summary of graph theory applications in chronic pain.

Of particular interest to studies of large scale network organization and precision neuromodulation is the concept of ***modularity***, which describes the functional segregation or clustering of nodes (i.e., brain regions) into subnetworks (i.e., *modules* or *communities*) that have stronger connections with nodes in the same module than with nodes in other modules (Figure 2F) (272, 274, 277, 280, 281). Community detection algorithms (e.g., Louvain, Infomap) are applied to thresholded network graphs to identify synchronous subnetworks characterized by many within-module connections and fewer between-module connections (281, 282). Modularity and community structure are universal organizing principles of complex biological systems (190, 275, 278, 281). Highly modular networks exhibit functional segregation, and subnetworks estimated from rsFC data reliably correspond with separate canonical RSNs involved in different aspects of cognition (e.g., somatosensory, limbic, attention, and default mode networks) (100, 101). Critically, network graphs produce valid and reliable estimates at the single subject level (23, 101, 283) and prior work has shown that *modularity* has prognostic value in predicting individual differences in treatment outcomes (284).

The decomposition of brain networks into functionally specialized modules enables the identification of highly connected *hub nodes* that are well situated to support integrative processing across the entire network (272, 280, 283). Hub nodes with high *participation coefficient (PC)*, also termed the “diverse club”, have the most diverse connectivity across the network's functional modules and are therefore well-suited to integrate activity between distinct subnetworks (280, 283, 285). Participation coefficient takes a maximal value for nodes that have an equal number of connections to each separate module in the network (Figure 2G). In humans, brain regions with hub-like characteristics facilitate integrative processing across distributed functional subnetworks and support modular network organization (285). Nodes with high PC also promote dynamic network reorganization to accommodate evolving task demands and their recruitment predicts task performance (286, 287). Damage to nodes with high PC impairs the modular architecture of human brain networks (288) and is a common predictor of disease severity across neuropsychiatric disorders ranging from Alzheimer's disease to chronic pain (289). Further, targeted disruption of network hubs with personalized fMRI-guided TMS markedly impairs cognitive and integrative function, providing additional causal evidence of the importance of hubs to large scale network integration (257).

Notably, whereas “hubs” of the pain matrix described in Section 2 are defined based on *a priori* functional attributions to implicated brain regions (i.e., regions of particular importance), hubs in a network graph are derived from the observed correlational structure of the studied network. There is substantial overlap between network hubs identified using data-driven community detection algorithms and putative Pain Matrix hubs, including ACC and aIns, which may reflect the fact that many pain-relevant regions are in higher-order association areas (280, 283). Importantly, the overlap between graph hubs and Pain Matrix hubs provides a viable alternative fifth network hypothesis with which to understand clinical stimulation effects at prior DBS and TMS targets. Because network “hubs” are more likely to be members of many distinct pain-relevant subnetworks, stimulating hub regions should have widespread effects on the multidimensional experience of chronic pain. Thus, we hypothesize that clinical benefit at any given target may reflect the “hub-ness” of the underlying target region.

### 5.1. Evidence for impaired network integration and hub disruption in chronic pain

The application of graph theoretical methods to chronic pain has begun to reveal widespread changes in network topology that are shared across pain etiologies but notably distinct from healthy populations (279). Pain networks defined based on intracranial physiologic responses to pain are inherently modular and segregate into subnetworks that reflect known pain processing domains (290). A variety of established graph theory terms describing network topology and the roles of individual nodes (e.g., Thalamus) have been shown to correlate with clinical pain metrics, including pain intensity, duration, and pain-related disability, suggesting that graph terms are behaviorally relevant (279).

Studies evaluating specific graph theory parameters across chronic pain conditions and species suggest that disruption of modular network organization is associated with emergence of the chronic pain state (291, 292). Direct comparisons of the full range of available graph terms between chronic pain populations and healthy cohorts and between different chronic pain conditions are limited by substantial variability in methods and reporting across the small number of available studies; however, there is an emerging consensus that global network changes and integrated local/global estimates like *participation coefficient* can more reliably distinguish patients with chronic pain from healthy controls. Specifically, chronic pain of various etiologies is associated with characteristic disruptions in the way that network *hubs* interact with multiple functional *modules*, leading to an emerging hypothesis that chronic pain is a state of dysfunctional network integration (291, 292). Global summary terms like the hub disruption index (HDI) and whole brain degree rank order disruption (*K*_D_) were developed to quantify how graphs constructed in individual subjects diverge from a normative comparison population. Consistent HDI and *K*_D_ changes have been observed in patients with chronic pain of various etiologies and in rodents with nerve-injury induced neuropathic pain, suggesting that, across species and pain conditions, well-connected regions become less well-connected in chronic pain. Overall, HDI and *K*_D_ analyses indicate that regions with hub-like characteristics (e.g., high participation coefficient or network centrality) are less hub-like, indicating that the emergence of chronic pain represents a fundamental shift in the identity of important network hubs within a modular network architecture. Taken together, prior work suggests that network-informed stimulation at personalized regions with hub-like characteristics should restore network integration and reduce chronic pain. However, applications of graph theory to clinical pain conditions are still in their infancy and it seems likely that a weighted combination of different graph parameters may be required to identify reliable stimulation sites in chronic pain (293).

## 6. Summary and future directions

The graph theory framework outlined above represents a fundamental reframing of precision medicine away from specific neuroanatomical targets defined at the population level toward data-driven, individualized local and global network estimates that are more reliably implicated in the pathogenesis of chronic pain. As with prior region and RSN models, combining network graphs with targeted stimulation can begin to support a causal link between the characteristics of specific network nodes (i.e., brain regions), global network organization, and the experience of chronic pain. We propose that studies of clinical neuromodulation should routinely employ pre/post brain imaging measures to facilitate network analysis and better ascertain mechanisms of clinically efficacious brain stimulation for individual patients, consistent with IMMPACT recommendations for clinical trials in chronic pain (57, 294, 295).

One key advantage of the graph theory approach over measurements of activity and connectivity derived from the BOLD signal is that it dramatically expands the types of information that can be estimated from a network. New applications of graph theory in network neuroscience are continually evolving. Whereas static network graphs provide a snapshot of network organization, recent advances in dynamic network analysis are beginning to show how network topologies evolve and change with learning and intervention (286, 296–298). Dynamic time-varying graph analyses may in turn facilitate a variety of analyses ranging from estimation of node promiscuity (i.e., membership in multiple distinct functional modules over time) to linking dynamic reorganization of networks to experiential states, such as high and low pain (99). Dynamic analyses may also be used to explore the state-dependence of neuromodulation targets depending on the context of stimulation, which builds upon the *who* and *where* questions of stimulation with additional information about *when* stimulation is most likely to be effective.

Associating specific network topologies with clinically-relevant states (e.g., high and low pain) will also facilitate more advanced analyses of network controllability, which describe how stimulating a subset of key nodes might drive a network toward a desired state (i.e., low-pain) (299–302). Such an approach could be undertaken through off-line identification of specific regions that reliably transition a given patient into a low pain state (i.e., identifying network characteristics of the pain-relieving node) or by recording real-time pain ratings during brain imaging to determine whether fluctuations in clinical pain are accompanied by temporally linked dynamic shifts in network organization. It is likely that combining these two levels of analysis will be required to build a comprehensive precision network model capable of predicting which regions are most likely to induce favorable state transitions for individual patients (e.g., from high to low pain).

Although graph theory has previously linked network reconfiguration dynamics with behavior following learning or intervention (284, 287), to our knowledge it has never been used to evaluate candidate brain regions for therapeutic stimulation. We hypothesize that deploying precision network neuroscience and graph theory to trials of therapeutic brain stimulation will enable a deeper understanding of the networks that contribute to the experience of pain and facilitate the identification of more effective personalized stimulation targets. The parallel emergence of valid and reliable methods for single subject fMRI and an expanding array of graph theory tools for network neuroscience offer great promise to advance our understanding of the network correlates of pain and relief. However, taking full advantage of the considerable opportunities presented by ongoing trials of neuromodulation for pain will require careful attention to methods for data collection, analysis, interpretation, and dissemination. To conclude, we offer recommendations for future neuromodulation research to facilitate network analyses and ongoing development of circuit-based treatments for pain.

## 7. Recommendations for future research

### 7.1. Data collection

1. Collect sufficient data to generate precision network estimates.
  a. At minimum, studies should acquire high resolution T1-weighted anatomical sequences for co-registration, diffusion tensor imaging (DTI) sequences for estimating structural white matter connections, and resting state fMRI (rs-fMRI) sequences for estimating functional brain networks, ideally at a field strength of 3 T (36, 57, 295). A discussion of the full range of sequences and parameters is beyond the scope of the present article, but we provide some “minimum necessary” acquisition guidelines below. We suggest that investigators establishing new imaging protocols follow guidelines from large consortia like the Adolescent Brain Child Development (ABCD) study, which has ready-to-use protocols for most Siemens, GE, and Philips scanners (303).
    i. Anatomical: 3D T1-weighted images with at least 1 mm × 1 mm × 1 mm resolution (approx. 5–7 min acquisition time).
    ii. White Matter (DTI): at minimum including one unweighted image (i.e., T2 or *b* = 0) and at least 6 diffusion-weighted images with orthogonally oriented gradients. We recommend using a high angular resolution diffusion imaging (HARDI) protocol with at least 60 orthogonal diffusion directions and multiple gradient strengths for improved white matter tract tracing performance (approx. 6–10 min acquisition time).
    iii. Functional MRI: Optimization of fMRI parameters often involves trade-offs between spatial and temporal resolution, with specific protocols customized to the population and brain regions of interest. Most contemporary fMRI protocols have spatial resolution of 2–3.5 mm^3^ and temporal resolution (defined by the TR, or repetition time, to acquire one full brain volume) of around 2 s (304). Precision network estimates require ∼45 min of data using conventional rs-fMRI sequences (i.e., TRs of 2–3 s) and ∼10–15 min using multi-band fMRI (which reduces TR to ∼800 ms) and multi-echo fMRI (which acquires up to 4 separate brain volumes per TR, each with different SNR characteristics) (23, 256).
2. Collect data over multiple sessions or during real-time symptom ratings.
  a. Linking experiential pain states with dynamic network graphs can improve understanding of brain-behavior relationships (99).

### 7.2. Data analysis

1. Follow consensus guidelines for data preprocessing and analysis to facilitate comparison with prior work (305–307).
2. Use appropriate parameters to generate valid network graphs.
  a. Use standard parcellations to define nodes (i.e., brain regions) that ensure adequate coverage of brain areas of interest and correspond with functional units of brain organization (101, 308).
  b. Apply rsFC thresholds to ensure that edges are sufficiently sparse to generate meaningful and interpretable graphs, typically including only the top 10% of connections (101, 278, 281).

### 7.3. Data interpretation

1. Follow consensus guidelines for reporting data acquisition and analysis parameters for functional brain imaging studies (309).
2. Ground interpretations in extant network models of pain perception such that each study can support or refute available hypotheses to guide community consensus (38, 43, 94, 115, 310).

## Data Availability

The original contributions presented in the study are included in the article, further inquiries can be directed to the corresponding author.
